# The gender health gap in Europe’s ageing societies: universal findings across countries and age groups?

**DOI:** 10.1007/s10433-020-00559-6

**Published:** 2020-02-17

**Authors:** Alina Schmitz, Patrick Lazarevič

**Affiliations:** 1 TU Dortmund, Emil-Figge-Str. 50, 44227 Dortmund, Germany; 2 Austrian Academy of Sciences, Vienna Institute of Demography, Vordere Zollamtsstraße 3, 1030 Vienna, Austria

**Keywords:** Health inequalities, Age differences, Cross-country comparison, Survey of Health, Ageing and Retirement in Europe (SHARE)

## Abstract

We provide a systematic country and age group comparison of the gender gap in several generic health indicators and more specific morbidity outcomes. Using data from the Survey of Health, Ageing and Retirement (SHARE), we examined the gender gap in the prevalence of poor self-rated health, chronic health conditions, activity limitations, multimorbidity, pain, heart attacks, diabetes, and depression in three age groups (50–64, 65–79, and 80+) based on linear probability models with and without adjustment for covariates. While women were typically disadvantaged regarding poor self-rated health, chronic health conditions, activity limitations, multimorbidity, pain, and depression, men had a higher prevalence of heart attacks and diabetes. However, the gender gap’s magnitude and sometimes even its direction varied considerably with some age trends apparent. Regarding some health indicators, the gender gap tended to be higher in Southern and Eastern Europe than in Western and Northern Europe. All in all, the presence of a gender health gap cannot be regarded as a universal finding as the gap tended to widen, narrow or even reverse with age depending on the indicator and country.

## Introduction

Because of rapid population ageing in Europe, there is an increasing interest in the health status of people at older ages and differences in the health status of women and men have attracted scholarly attention for a long time. Empirical results on gender inequalities in health may appear puzzling as the direction of the gender health gap depends on the indicator. The prevalence of potentially lethal diseases, like coronary heart diseases, stroke, or lung cancer, is higher in men (Read and Gorman 2011; Oksuzyan et al. 2018). Accordingly, women outlive men for several years in all countries of the European Union (European Commission 2015). In contrast, more women report non-fatal but debilitating conditions, such as arthritis, depression, and cognitive impairment (Crimmins et al. 2010; Leveille et al. 2000; Schmitz and Brandt 2019). For self-rated health (SRH), results are less uniform, but many studies report a female disadvantage which is attenuated or even reversed when other health indicators are controlled for (Crimmins et al. 2010; Bardage et al. 2005).

While some scholars draw conclusions like ‘women are sicker, but men die quicker’—the so-called male–female health survival paradox (Oksuzyan et al. 2008)—these ‘taken-for-granted assumptions’ have been criticised by others (Lahelma et al. 2001) as the magnitude of the gender health gap differs remarkably depending on the health indicator and country under study. For the age group 50+, Crimmins et al. (2010) examined gender differences in functional limitations, disability, disease prevalence, and SRH. They conclude that even though there is a remarkable consistency in the direction of gender differences, their magnitude shows enormous variation between countries. This holds true for indicators like SRH and depression along with more objective indicators, such as activity limitations and chronic diseases like arthritis. Recent studies confirmed this regional variation of the magnitude of the gender gap in frailty and activities of daily living in the older population (Ahrenfeldt et al. 2019; Scheel-Hincke et al. 2019). Furthermore, there are significant health differences between age groups and birth cohorts (Lahelma et al. 2001; Bird et al. 2012; Macintyre et al. 1996). Thus, differences in women’s and men’s morbidity profiles are neither a universal finding across age groups and health indicators nor the magnitude of the gender health gap invariant across countries and birth cohorts (Bird et al. 2012).

## (Social) Explanations for gender inequalities in health

There are mainly three explanatory approaches for gender differences in health: biological, methodological, and social explanations. While biological explanations focus on the role of sex hormones and other differences in physiological systems, methodological explanations assume that women are more likely to seek medical advice, report health problems in interview situations, and report differentially when seeking help (Acciai and Hardy 2017). Studies that empirically test such claims are scarce and the few existing studies focusing on well-being and depression provided no evidence supporting this hypothesis (Acciai and Hardy 2017; Oksuzyan et al. 2019). Furthermore, gender stereotypes might influence the practice of medical diagnosis and the expression of symptoms by women and men (Sen et al. 2002). It has also been argued that men tend to use health services less frequently than women, possibly resulting in an underdiagnosis of certain diseases. However, it has been shown that gender differences in doctor consultations are attenuated or even reversed when overall health or the severity of health conditions is considered (Roy and Chaudhuri 2012; Courtenay 2000).

Social explanations highlight the importance of social determinants of health—i.e. the conditions in which people are born, grow, live, work, and age. One key social determinant of health is *gender*, that is, ‘the socially constructed roles, behaviour, activities, and attributes that a particular society considers appropriate for men and women’ (WHO 2019). Gender roles affect the way women and men engage in education, the labour market, domestic and care work, and health behaviours (Loretto and Vickerstaff 2015; Courtenay 2000; Haberkern et al. 2015). Additionally, they affect the design of formal institutions and policies, constraining or incentivising individuals’ choices throughout the life course (Bonsang et al. 2017; Bird and Rieker 2008). Social explanations for gender inequalities in health stress the relevance of *health behaviours*, such as tobacco and alcohol consumption, dietary habits, physical activity, and healthcare utilisation (Oksuzyan et al. 2010; Mahalik et al. 2007; Luy 2003), *socio*-*economic factors*, such as financial resources and working conditions (Read and Gorman 2011), and *psychosocial factors*, such as critical life events, social network characteristics, and coping styles (Thoits 2011; Lachman et al. 2011). From this point of view, gender differences in health arise from a gendered access to protective resources (e.g. education, income, and social support) and a differential exposure to health risks (e.g. occupational hazards, family responsibilities like caring for older relatives, and unhealthy behaviours).

Recent studies have revealed considerable differences between European societies regarding gendered patterns of family responsibilities (Schmid et al. 2012; Brandt 2013) and labour market participation (Cipollone et al. 2012; Edge et al. 2017) so that country differences in the gender health gap come with little surprise. Further, gender differences in health promoting resources and health risks do not only vary depending on the country context, but are also likely to differ between age groups and people of different birth cohorts. Thus, separate analyses by age groups are necessary—also because aggregated data over all age groups would bias results towards younger ages due to their typically greater number of cases. Still, most studies on gender differences in health do not differentiate between age groups so that between-age-group variation remains hidden. Our study provides an analysis of the gender health gap in old age for several generic health indicators and more specific morbidity outcomes. We compare the health status of women and men between 16 European countries stratified by age both unadjusted and adjusted for relevant socio-demographic characteristics.

## Data and methods

### Study population and sample

Data were drawn from the sixth wave of the *Survey of Health, Ageing and Retirement in Europe* (SHARE), a cross-country comparable and nationally representative study on health and social conditions of persons aged 50 years and older living in private households. A detailed description of the survey methodology has been provided elsewhere (Börsch-Supan et al. 2013; Munich Center for the Economics of Aging 2018). We grouped the sample into three age groups (50–64, 65–79, and 80+). Our analysis covers 16 countries, including Northern Europe (Denkmark and Sweden), Eastern Europe (Croatia, Czech Republic, Estonia, Poland, and Slovenia), Southern Europe (Greece, Italy, and Spain), and Western Europe (Austria, Belgium, France, Germany, Luxembourg, and Switzerland). We excluded Portugal from our analyses due to small sample sizes in older age groups. In total, 8252 respondents were excluded due to missing values for at least one of the variables of interest. The final sample included 55,446 participants.

The sample’s mean age amounted to 65.5 years (Table A1 in the Appendix). Around 51% of the samples belonged to the age group 50–64, these aged 65–79 comprised another third (37%), and around 12% of the study participants are aged 80+. This highlights the necessity for age stratification to unveil potential age-specific differences since the weight of the respondents aged 50–64 in the sample would mask any differences in older age groups. When comparing age groups, the share of women increased with age in every country and amounted to around 63 per cent in the age group 80+ in the overall sample—probably due to men’s lower life expectancy.

### Variables

To present a broad overview of health differences, this study uses a diverse set of health indicators, namely SRH, chronic health conditions, the global activity limitation indicator (GALI), multimorbidity, the presence of pain, and the prevalence of heart attacks, diabetes, and depression. To compare the results between health indicators, we dichotomised all variables.

### Self-rated health

SRH represents a subjective overall evaluation of a person’s health status which is highly correlated with several other health indicators and predicts future morbidity, functional limitations, and mortality (Jylhä 2009). SRH was assessed by asking ‘*Would you say your health is…excellent/very good/good/fair/poor?*’. A binary variable was coded into ‘good health’ (good or better) versus ‘poor health’ (fair or poor).

### Chronic health conditions

Respondents were asked a global chronic morbidity question: ‘*Some people suffer from chronic or long*-*term health problems. By chronic or long*-*term we mean it has troubled you over a period of time or is likely to affect you over a period of time. Do you have any such health problems, illness, disability, or infirmity?*’ This indicator is, like SRH, part of the ‘Minimum European Health Module’ (MEHM) that is used in multiple administrative surveys to shortly assess the respondent’s health status (Robine and Jagger 2003) and is further used to calculate healthy life expectancy (Jagger et al. 2008).

### Global activity limitation indicator

GALI is a comprehensive indicator that has proved to appropriately reflect health-based activity limitations in cross-country comparisons (van Oyen et al. 2006; Jagger et al. 2010). It was collected by the question: *‘For the past* 6 *months at least, to what extent have you been limited because of a health problem in activities people usually do?’ (not limited/limited but not severely/severely limited).* We dichotomised the variable into ‘no limitations’ versus ‘limitations’.

### Multimorbidity

Multimorbidity, the presence of several diseases, is a common health condition in old age and associated with an elevated risk of mortality, functional limitations, and reduced quality of life (Marengoni et al. 2011). In accordance with other studies (see the systematic review by Violan et al. (2014), we defined multimorbidity as reporting diagnoses of at least two diseases or chronic health conditions (heart attack or any other heart problem; high blood pressure or hypertension; high blood cholesterol, stroke or cerebral vascular disease; diabetes or high blood sugar; chronic lung disease; cancer; stomach/duodenal ulcer; Parkinson disease; cataracts; hip fracture or other fractures; dementia, organic brain syndrome or any other serious memory impairment; other affective or emotional disorders; rheumatoid arthritis, osteoarthritis, other rheumatism; chronic kidney disease).

### Presence of pain

Chronic pain is a highly salient health condition with immense psychosocial consequences as it impairs a person’s well-being and the ability to maintain an independent lifestyle (Breivik et al. 2006). According to the question ‘*Are you troubled with pain?*’, we grouped the respondents in those reporting or not reporting the presence of pain.

### Chronic diseases

Additionally to multimorbidity, we analysed several chronic diseases which are among the leading causes for disability worldwide (Murray and Lopez 2013). These were diagnosed heart attacks (including myocardial infarction, coronary thrombosis, or any other heart condition including congestive heart failure; shortened to ‘heart attacks’ below), diagnosed diabetes or high blood sugar (shortened to ‘diabetes’), and a high probability of depression according to the EURO-D scale, consisting of twelve items on depressive symptoms. In accordance with other studies, we chose a cut-off point of four symptoms to identify respondents with a high risk of depression (Prince et al. 1999).

### Covariates

To account for some of the social determinants of health, we adjusted the estimated gender gaps for several covariates: educational attainment according to the International Standard Classification of Education (UNESCO 1997) with seven categories, marital status (married/registered partnership, divorced/living separated from spouse, never married, widowed), whether their household was able to make ends meet (four categories) or would be able to afford an unexpected expense without borrowing money (yes/no) as assessed by the household head, rural (small town/rural area/village) versus urban (big city, suburbs/outskirts of a big city, or large town) residential area, and the number of doctor’s visits during the last year (0–1/2–3/4–9/10+). To account for potentially nonlinear relationships between these variables and the probability of a health condition and due to the variables’ scaling, we used all covariates’ categories as sets of dummy variables.

### Statistical analysis

We computed country-specific gender gaps via linear probability models (LPM) separately for respondents in the age groups 50–64 years, 65–79 years, and 80+ (i.e. three models per country). Using this approach, the gender coefficients represent the percentage difference in the prevalence of the corresponding health condition by gender in each age group. In a second step, we adjusted these gender gaps for potentially important covariates to account for gender differences in some social determinants of health (i.e. six models per country overall).

In all following analyses, positive estimates represent a higher prevalence of the health problem in women. We weighted all analyses with the calibrated cross-sectional weight provided by SHARE to account for country-specific sampling strategies and national differences in response rates.

## Results

### Overall prevalence of health conditions

Table 1 presents the overall prevalence of the health conditions separately by gender and age. Generally, health conditions were more prevalent in the older age groups. The prevalence of poor SRH, chronic health conditions, activity limitations, multimorbidity, pain, and depression was greater in women in every age group as compared to men. In contrast, heart attacks were more prevalent in men of all age groups as well as diabetes.

**Table 1:** Prevalence of health conditions in the pooled sample by age (in %)

	Men			Women			Total
	50–64	65–79	80+	50–64	65–79	80+	
Poor self-rated health	29.00	39.51	56.05	28.76	45.03	66.58	38.01
Chronic health conditions	43.80	52.90	62.26	44.56	55.97	68.01	50.64
Activity limitations	34.72	44.70	62.85	36.89	51.10	73.14	44.46
Multimorbidity	32.98	53.10	62.62	35.53	59.43	72.82	46.69
Presence of pain	36.49	39.83	47.55	45.95	56.56	67.62	46.44
Heart attacks	8.23	16.17	24.03	3.46	10.68	18.88	10.32
Diabetes	10.08	18.33	18.21	6.46	15.03	16.56	12.36
Depression	19.01	19.88	30.36	33.38	36.56	46.95	29.10

*Source*: SHARE wave 6, release 7.0.0. Author’s calculations. All analyses were weighted

### The gender health gap by countries and age groups

In the following graphs, the gender gap is depicted with 95%-CI separately for the three age groups within each country. For every subgroup, there are two estimates showing the overall gender gap and the gender gap adjusted for covariates.

### Poor self-rated health

**Fig. 1:**
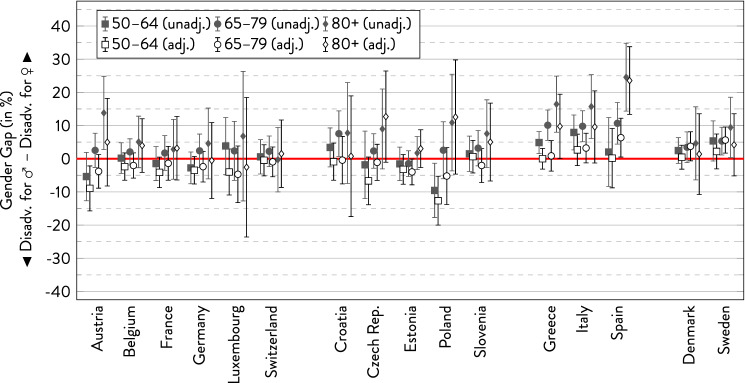
Gender gap in poor self-rated health across age groups by country. *Source*: SHARE wave 6, release 7.0.0. Author’s calculations. All analyses were weighted

As Fig. 1 shows, more women than men reported poor health across most age groups in Southern and Northern Europe—at least in the unadjusted models where the gender gaps were greatest in the Southern European countries, but rather small in Northern Europe. In Western and Eastern Europe, the picture was more complex, and the direction of the gender gap within countries partly varied between age groups. Yet, when adjusting for socio-demographic characteristics, the gender gap was rather small in Northern and Southern Europe (with the exception of the oldest age group). In some of the Eastern and Western European countries, adjusting for covariates even reversed the gender gap to the disadvantage of men. However, these nominal gaps should be cautiously interpreted, as coefficients were not statistically significant in most cases. Typically, there was an age trend with increasing gender gaps to women’s disadvantage, with outstandingly large gender gaps in the oldest age group in Spain.

### Chronic health conditions

**Fig. 2:**
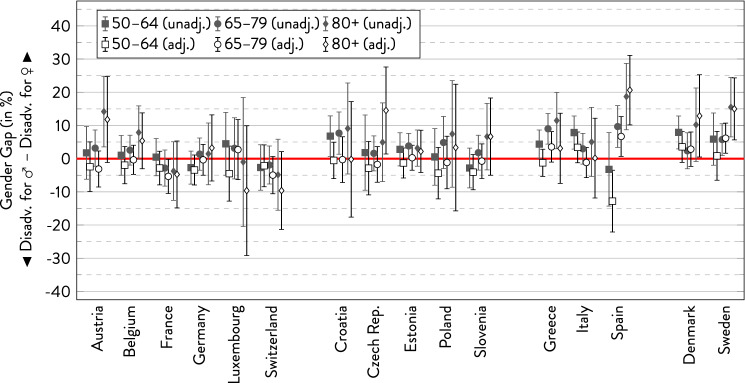
Gender gap in chronic health conditions across age groups by country. *Source*: SHARE wave 6, release 7.0.0. Author’s calculations. All analyses were weighted

For the presence of any chronic health condition, results were similar to the findings for SRH (Fig. 2). While more women reported health conditions in most age groups in Southern and Northern Europe, gender differences were less uniform in Western and Eastern Europe. Except for the older age groups, gender differences were relatively small in all European regions. Outstandingly large gender gaps were, again, observed in the oldest age group in Spain. Where there were rather consistent age trends, the gender gap typically increased to the disadvantage of women, whereas it reversed to the disadvantage of men in France and Switzerland. However, the large confidence intervals highlight the uncertainty of these trends. In other countries, age trends were inconsistent, non-existent, or the gap narrowed at older ages. Adjusting for covariates typically decreased the gender gap or even reversed gender differences to the disadvantage of men.

### Activity limitations

**Fig. 3:**
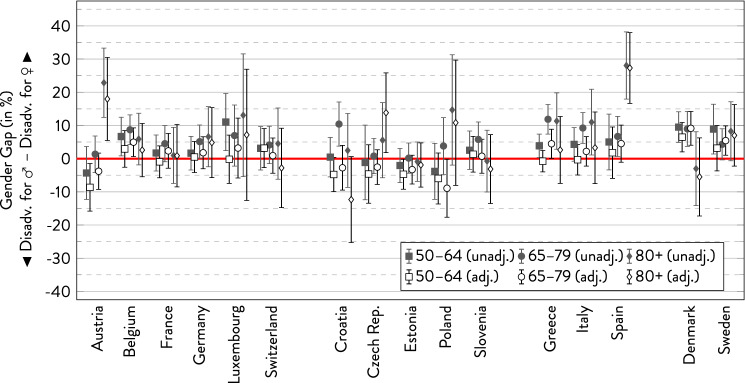
Gender gap in activity limitations across age groups by country. *Source*: SHARE wave 6, release 7.0.0. Author’s calculations. All analyses were weighted

In the unadjusted models, there was a greater prevalence of activity limitations in women than men in most age groups except in Eastern Europe (Fig. 3). In the few cases where the gender gap was at the expense of men, the gap was relatively small and not statistically significant. The gender gap proved particularly pronounced in the oldest age group in Austria and Spain. When comparing age groups, the gender gap increased to the disadvantage of women at older ages in Austria, Germany, the Czech Republic, Poland, and the Southern European countries. Denmark was the only country where it reversed to the disadvantage of men in the oldest age group. In the other countries, there was no clear age trend. Thus, the gender gap in activity limitations overall tended to increase if it changed at all. Regional differences were less pronounced than for SRH and chronic health conditions. Again, controlling for covariates typically reduced the gender gap in most cases and sometimes even reversed it to the disadvantage of men, although not with any statistically significance.

### Multimorbidity

**Fig. 4:**
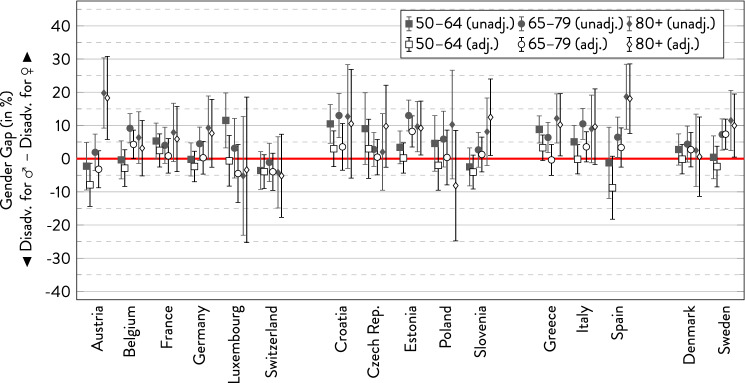
Gender gap in multimorbidity across age groups by country. *Source*: SHARE wave 6, release 7.0.0. Author’s calculations. All analyses were weighted

Without adjustment for covariates, there was a (nominally) higher prevalence of multimorbidity in women of most age groups in almost every country (Fig. 4). Outstandingly large gender gaps were observed in the oldest old in Austria and Spain. Notable counterexamples were found in some age groups in Austria, Luxembourg, Switzerland, and Slovenia—showing a (slightly) higher prevalence of multimorbidity in men. However, the gender gaps were statistically insignificant in most countries and age groups. In most countries with rather consistent age trends, the gender gap in multimorbidity was more pronounced at the expense of women in the older age groups (Austria, Germany, Poland, Slovenia, Italy, Spain, and Sweden). In contrast, the gap reversed to the disadvantage of men in Luxembourg. In the remaining countries, age differences were inconsistent. Regarding the countries geographical location, the gender gap tended to be relatively small in Northern Europe. Adjusting for covariates did again not significantly change the results. As before, if there were larger differences between the unadjusted and the adjusted model, they typically reduced the gender gap or even reversed the gender gap to the disadvantage of men.

### Presence of pain

**Fig. 5:**
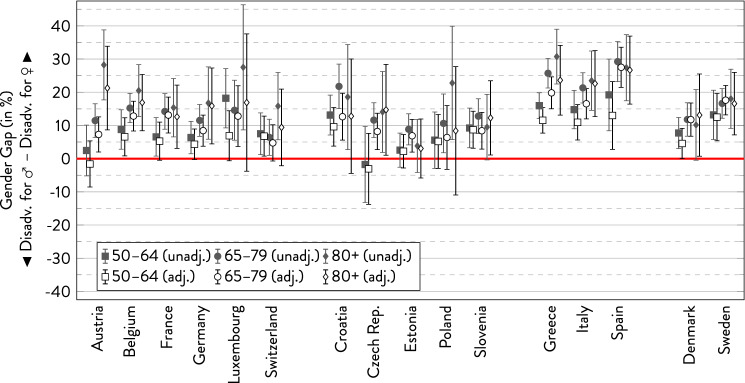
Gender gap in the presence of pain across age groups by country. *Source*: SHARE wave 6, release 7.0.0. Author’s calculations. All analyses were weighted

Across most countries and age groups, there were strong and statistically significant gender gaps in the presence of pain at the expense of women (Fig. 5). In countries with consistent age trends, gender gaps tended to increase with age with the largest gaps in the oldest age group in Austria, Luxembourg, Poland, Greece, and Spain. While there were some inconsistent age trends, a reversing gender gap at older ages to the disadvantage of men did not occur. The only apparent regional similarity was that the gender gap in pain was most pronounced in Southern European countries where it also more often tended to increase with age. Like before, if there were notable differences, adjusting for covariates typically reduced the gender gap in pain, albeit not statistically significantly.

### Heart attacks

**Fig. 6:**
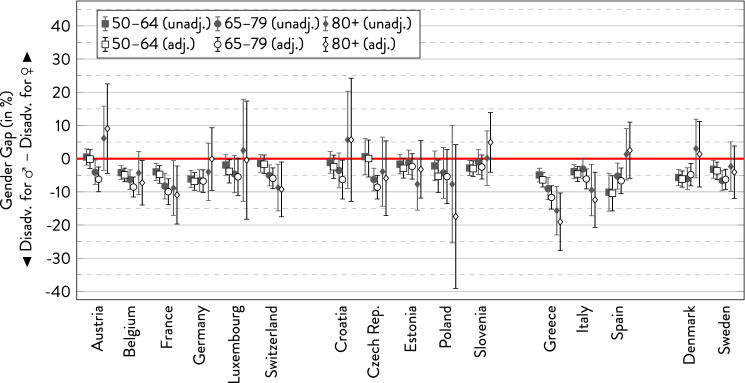
Gender gap in heart attack across age groups by country. *Source*: SHARE wave 6, release 7.0.0. Author’s calculations. All analyses were weighted

As displayed in Fig. 6, the prevalence of heart attacks was generally higher in men than in women in almost all countries—at least in the younger age groups. However, the gender gap was overall smaller than for the other indicators. Especially in the youngest age group, the gender gap was rather small, whereas differences were increasing in the older age groups in some countries at the expense of men (France, Switzerland, Estonia, Poland, Greece, and Italy). In contrast, the gender gap was narrowing in Slovenia, Spain, and Denmark. In all other countries, age trends were either inconsistent or non-existent. Regarding countries’ geographical location, the gender gap in heart attack prevalence was especially small across all age groups in some countries of Eastern Europe and more pronounced in Southern Europe, especially in Greece. Adjusting for covariates did not affect the gender gap strongly with the notable exception of changing the gap to the disadvantage of women in the oldest age group in Austria and Slovenia and reinforcing the gender gap to men’s disadvantage in the oldest old in Poland.

### Diabetes

**Fig. 7:**
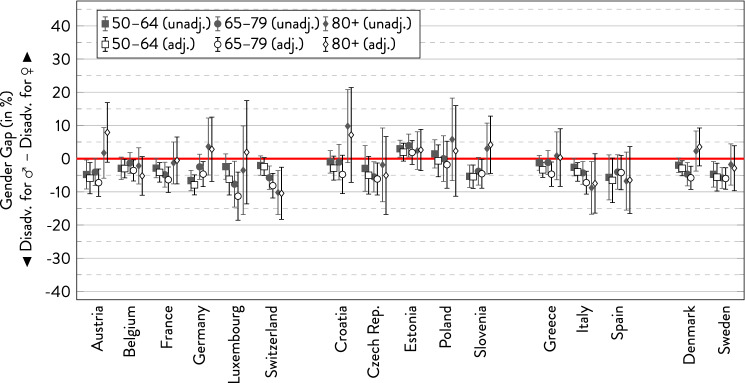
Gender gap in diabetes across age groups by country. *Source*: SHARE wave 6, release 7.0.0. Author’s calculations. All analyses were weighted

Looking at the gender gap in diabetes, the prevalence was greater in men than in women in most cases (Fig. 7). Similar to the findings on heart attacks, the gender gap in diabetes was rather small and not statistically significant in most countries. There was a reversing gap to the disadvantage of women in the oldest age group in some countries, whereas the gender gap increased at the expense of men only in Switzerland and Italy. Only minor and inconsistent age differences between age groups were observed in the remaining countries. As for geographical location, a gender gap nominally to the disadvantage of women was observed more often in the Eastern European countries. As with heart attacks, adjusting for socio-demographic variables hardly changed the results in most cases with a slight tendency of increasing the disadvantage for men.

### Depression according to EURO-D

**Fig. 8:**
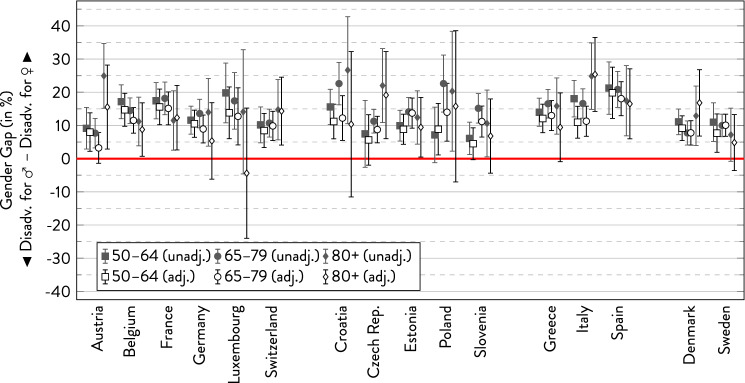
Gender gap in depression across age groups by country. *Source*: SHARE wave 6, release 7.0.0. Author’s calculations. All analyses were weighted

Of all health indicators considered in our analysis, Fig. 8 documents that depression showed the clearest gender health gap across all countries and age groups. There were only few non-significant gender gaps in the younger and in the oldest age group (however, in part due to large confidence intervals). Regarding age trends, there were some countries with a nominal increase to the disadvantage of women, some with a decrease in the gap in favour of women, and also countries with no apparent systematic changes. When looking at regional similarities, the gender gap in depression tended to be highest in Southern Europe across all age groups and relatively small in Northern Europe. As for the other indicators, but more strongly, adjusting for covariates decreased gender gaps, leading to insignificant differences between woman and men especially in the oldest age group.

## Discussion

To the best of our knowledge, our study is the first to examine the gender health gap across different age groups in Europe’s ageing societies including a wide range of health indicators. In the pooled SHARE sample, without and with adjustment for covariates, women displayed a worse health status with respect to most of the health indicators with only the prevalences of heart attacks and diabetes being higher in men.

### Gender health gap: universal findings across countries and age groups?

Whereas our analyses support the findings of a range of studies showing a female excess in depression (Acciai and Hardy 2017) and pain (Bartley and Fillingim 2013), the existence of a gender gap cannot be taken for granted for most of the other health indicators: Our results do not universally reflect a female excess in activity limitations and multimorbidity found in many other studies (see the overviews in Leveille et al. 2000 for activity limitations and Violan et al. 2014 for multimorbidity). In a similar vein, the often stated male predominance in heart attacks (Oksuzyan et al. 2018) was not confirmed in all age groups and countries. However, discrepancies in the results might also stem from differences in the operationalisation of health indicators, study populations, and statistical methods.

Furthermore, the gender gap’s magnitude was small in many age groups across countries and often statistically not significant, so that there might be no actual gender gap. All in all, we conclude that the gender health gap cannot be regarded as ‘given’ in Europe’s ageing populations. Instead, the direction and the magnitude of health inequalities between women and men strongly depend on the health indicator, age group, and country under study. Even some of the ‘taken-for-granted assumptions’ (e.g. women rate their health worse than men do; more men suffer from potentially lethal conditions like heart attacks) are not universally reflected by our empirical results.

Further, our results clearly demonstrate the necessity of differentiating between age groups when comparing women’s and men’s morbidity profiles as the gender gap in SRH, activity limitations, multimorbidity, pain, and depression was mostly increasing with age at the expense of women. In contrast, gender differences in heart attacks were widening to the disadvantage of men in older age groups in some countries. Concerning diabetes, which was more common in men than women in the younger age groups, the gender gap reversed in old age. For chronic health conditions and depression, no clear age trends were identified.

Last but not least, some regional similarities were identified. The gender gap in heart attacks was relatively small in Eastern Europe as compared to other regions. Southern Europe stood out with comparably large gaps to the disadvantage of women for poor SRH, activity limitations, pain, and depression. In Northern Europe, gender differences in multimorbidity, depression, and SRH were rather small as compared to the other regions.

### Underlying mechanisms of the gender health gap and implications for future research

While biological factors such as genetic, anatomical, and endocrine characteristics might explain gender differences in health to some extent, biological explanations cannot explain the variations in the gender health gap between countries that we found in our study. Social explanations of the gender gap in health focus on gender differences in socio-economic status, psychosocial characteristics, and health behaviours. It is likely that the relevance of these explanations depends on the health indicator under study: For example, whereas the gender gap in chronic diseases like diabetes might be largely explained by gender differences in dietary habits and physical activity, these factors are certainly less relevant with regard to the gender gap in depression for which a complex interplay of social and biological factors has been proposed (Kuehner 2017). For generic health indicators like SRH, activity limitations, multimorbidity, and pain, disentangling the underlying mechanisms of the gender health gap promises to be an even greater challenge.

Overall, our results support the relevance of social explanations for the gender health gap as it was typically reduced or even reversed to the advantage of women when controlling for covariates that represented social determinants of health. Since women—at least in older birth cohorts—are typically disadvantaged in terms of resources such as education and income, a reduction in the gender gap by including these covariates fits these explanations. However, as the gender gaps did not fully vanish when controlling for covariates, it seems likely that these explanations are not solely responsible for the health differences between women and men. Yet, acknowledging the influence of social determinants of health, as demonstrated in this paper, provides a starting point for policy makers to reduce gender differences in health. For some health indicators, we observed a greater gender health gap to the disadvantage of women in Southern Europe, while gender inequalities were less pronounced in the Northern European countries. These findings are in line with studies demonstrating that greater gender equality—typically found in the Nordic social democratic welfare regimes—has a positive health effect on the health of women (King et al. 2018; Borrell et al. 2014).

Finally, we conclude that gender differences in health in one age group and country are not necessarily generalisable to other age groups and countries so that periodic re-examinations of the gender health gap are mandatory in order to develop and monitor targeted interventions for reducing health inequalities between women and men. Furthermore, our results suggest that gender differences in health should be considered when reforming labour market and healthcare policy in Europe’s ageing societies. For example, in the field of policies for extending the working life, there is a tendency to think of the end of working life as gender neutral or following a typical male trajectory (Loretto and Vickerstaff 2015). This falls short as poor health, whose likelihood differs by gender, is the most important barrier to extended working lives, and also the nature of work itself differs by gender (Edge et al. 2017).

### Limitations

There are several limitations of our study. First, our analysis is based on self-reported health measures which bear the risk of bias due to systematic differences in reporting styles of people of different age groups, educational and cultural backgrounds (Jürges 2007). While it is likely that health indicators which are based on a subjective evaluation (e.g. SRH) are influenced by such reporting differences (Lazarevič 2018), this bias might be of less relevance for indicators such as diagnosed diseases. Still, even results on diagnosed diseases might be biased due to differences in the healthcare utilisation of women and men (for diabetes, see Cowie et al. 2006 or a differential chance of survival; for heart attacks, see Sun et al. 2018). Furthermore, our study was limited to the countries included in SHARE, so that regional differences should be interpreted with caution. Further analyses incorporating other or even more countries are desirable. It must also be mentioned that the confidence intervals of the gender gaps were often relatively large due to small sample sizes or a low prevalence of certain health conditions in some age groups. In addition, the study sample consists of people living in private households. As the likelihood of older adults being institutionalised varies across countries with different long-term care options, it must be kept in mind that older adults with poor health remain in their households in some countries, whereas their counterparts in other countries live in institutions. An analysis of the gender health gap in institutionalised individuals, while not possible with the data used here, would be highly desirable.

Finally, yet importantly, differences at the level of the overall population can, as this study has shown with regard to age and country, mask considerable inequalities within the aggregate. A disadvantage of one group in the whole population with respect to a certain health indicator does not necessarily imply that all subgroups of women and men face this health disadvantage (Schofield 2012). Hence, more studies investigating the gender health gap in different population groups (e.g. between different socio-economic groups) are needed to evolve our understanding of the relationship between gender and health.

These shortcomings, however, do not challenge our main conclusion that the relationship between gender and health defies easy summary as even some of the ‘taken-for-granted assumptions’ were not completely supported in our analysis of the gender health gap in Europe’s ageing populations.

## Appendix

**Table A1:** Sample characteristics by country

	*N*	Age		Share of age groups (in %)			Share of women by age groups (in %)			
		Mean	SD	50–64	65–79	80+	50–64	65–79	80+	Total
Austria	2835	66.31	10.13	50.40	36.87	12.73	53.09	55.13	63.88	55.21
Belgium	5147	65.14	10.68	52.26	35.23	12.52	50.33	52.69	60.99	52.49
France	3505	65.40	10.94	51.58	34.89	13.53	51.01	54.91	63.71	54.09
Germany	4075	66.02	10.29	49.99	37.91	12.11	51.21	53.29	64.59	53.62
Luxembourg	1419	63.87	10.22	56.93	32.76	10.31	50.68	51.67	60.23	51.99
Switzerland	2493	65.94	10.01	51.07	36.00	12.93	51.14	53.06	59.45	52.91
Croatia	2298	65.58	9.85	51.73	37.48	10.79	52.11	55.94	63.60	54.79
Czech Rep.	4014	64.73	9.59	51.59	39.09	9.32	52.60	54.50	67.36	54.72
Estonia	4574	66.70	10.48	46.34	39.74	13.93	56.75	64.06	73.70	62.02
Poland	1462	63.73	9.92	59.51	31.82	8.67	50.89	61.47	72.19	56.10
Slovenia	3716	65.12	10.25	53.55	35.23	11.22	50.05	55.64	69.68	54.22
Greece	4371	66.09	10.74	49.29	36.04	14.67	52.46	53.06	56.21	53.23
Italy	4454	65.69	10.61	48.04	39.20	12.76	51.92	53.56	60.96	53.72
Spain	4264	65.28	10.54	53.13	34.49	12.38	50.88	54.84	60.57	53.45
Denmark	3446	65.20	10.06	50.29	39.50	10.20	51.02	51.22	63.54	52.37
Sweden	3373	67.00	10.18	44.73	41.19	14.08	49.80	51.74	61.20	52.20
Total	55,446	65.51	10.47	51.19	36.57	12.24	51.29	54.47	63.23	53.91

*Source*: SHARE wave 6, release 7.0.0. Authors’ calculations. All analyses were weighted
